# Structural Insight Into the SARS-CoV-2 Nucleocapsid Protein C-Terminal Domain Reveals a Novel Recognition Mechanism for Viral Transcriptional Regulatory Sequences

**DOI:** 10.3389/fchem.2020.624765

**Published:** 2021-01-12

**Authors:** Mei Yang, Suhua He, Xiaoxue Chen, Zhaoxia Huang, Ziliang Zhou, Zhechong Zhou, Qiuyue Chen, Shoudeng Chen, Sisi Kang

**Affiliations:** ^1^Guangdong Provincial Key Laboratory of Biomedical Imaging, Molecular Imaging Center, The Fifth Affiliated Hospital, Sun Yat-sen University, Zhuhai, China; ^2^Molecular Imaging Center, The Fifth Affiliated Hospital, Sun Yat-sen University, Zhuhai, China

**Keywords:** COVID-19, coronavirus, SARS-CoV-2, nucleocapsid protein, C terminal domain, crystal structure, transcription regulating sequences

## Abstract

Coronavirus disease 2019 (COVID-19) has caused massive disruptions to society and the economy, and the transcriptional regulatory mechanisms behind the severe acute respiratory syndrome coronavirus 2 (SARS-CoV-2) are poorly understood. Herein, we determined the crystal structure of the SARS-CoV-2 nucleocapsid protein C-terminal domain (CTD) at a resolution of 2.0 Å, and demonstrated that the CTD has a comparable distinct electrostatic potential surface to equivalent domains of other reported CoVs, suggesting that the CTD has novel roles in viral RNA binding and transcriptional regulation. Further *in vitro* biochemical assays demonstrated that the viral genomic intergenic transcriptional regulatory sequences (TRSs) interact with the SARS-CoV-2 nucleocapsid protein CTD with a flanking region. The unpaired adeno dinucleotide in the TRS stem-loop structure is a major determining factor for their interactions. Taken together, these results suggested that the nucleocapsid protein CTD is responsible for the discontinuous viral transcription mechanism by recognizing the different patterns of viral TRS during transcription.

## Introduction

The coronavirus disease 2019 (COVID-19) pandemic, caused by severe acute respiratory syndrome coronavirus 2 (SARS-CoV-2), has led to a total of 45,140,131 confirmed cases and 1,182,747 deaths across 216 countries and regions as of October 31, 2020 (World Health Organization, https://covid19.who.int). Despite remarkable efforts to study the pathological roles of the SARS-CoV-2 virus, there are still many mysteries about the life cycle of SARS-CoC-2.

Similar to other pathogenic betacoronaviruses (order *Nidovirales*, family *Coronaviridae*, subfamily *Coronavirinae*), SARS-CoV-2 was discovered as an enveloped, positive-sense, single-stranded RNA virus ~30 kilobase in genomic size (Wu et al., 2020; Zhou et al., 2020). Coronaviruses (CoVs) have the largest genomes among the RNA virus families and a conserved 5′ leader sequence (Lai and Stohlman, 1981; Sola et al., 2015). In the viral life cycle, the positive-sense RNA genome is replicated and transcribed by the viral RNA-dependent RNA polymerase (RdRp) protein (Sola et al., 2015; Snijder et al., 2016). The replication of the genome requires continuous RNA synthesis since a full-length complementary negative-strand (–) RNA is used as the template for the production of genomic RNA (gRNA) copies. In contrast, CoV transcription requires a unique discontinuous synthesis of (–) subgenomic RNA (sgRNA). The RdRp complex utilizes the template switching mechanism of the nascent (–) RNA fused with the genomic 5′ leader sequence to generate a nested set of subgenomic mRNAs (sgmRNAs) that are identical to the 5′ and 3′ termini of the viral genome(Pasternak et al., 2006; Sawicki et al., 2007). The discontinuous transcription process is controlled by a conserved transcriptional regulatory sequence (TRS), which is located after the conserved 5′ leader sequence (termed leader TRS, TRS-L) and in front of each ORF gene (termed body TRS, TRS-B). A prevailing model suggests that base pairing between the TRS-L and the complementary TRS-B occurs during (–) strand RNA synthesis(Sola et al., 2015). The pairing leads to template-switching events that generate discontinuous (–) strand RNAs, which serve as templates for the transcription of large amounts of discontinuous nested (+) strand sgmRNAs. These sgmRNAs encode conserved structural proteins (spike (S), envelope (E), membrane (M), and nucleocapsid proteins) and several accessory proteins (Sola et al., 2015; Wu et al., 2020). The SARS-CoV-2 nucleocapsid protein consists of five domains: an N-terminal tail region (residues 1 to 40), an N-terminal RNA binding domain (residues 41 to 173, termed N-NTD), a Ser/Arg-rich linker region (residues 174 to 249, termed LKR), a C-terminal dimerization domain (residues 250 to 364, termed N-CTD), and a C-terminal intrinsically disordered region (residues 365 to 419, termed IDR) (Supplementary Figure 1A).

According to a recent transcriptome study, Vero cells infected with SARS-CoV-2 produce 92.6% canonical full-length gRNA and nine sgRNAs, as well as 7.4% other non-canonical transcripts because of numerous discontinuous transcription events (Kim et al., 2020). Among the top transcribed sgRNAs, the sgRNA-encoded nucleocapsid is the most abundant transcript. Of note, a previous study suggested that the coronavirus nucleocapsid protein participates in the discontinuous transcription process of sgRNAs since depletion of the nucleocapsid-encoded region from the replicon reduces the synthesis of sgmRNAs but not gRNAs (Zúñiga et al., 2010). Importantly, the phosphorylation of the SARS-CoV nucleocapsid results in the recruitment of the RNA helicase DDX1 and then enables the transcription in the transition from a discontinuous process to a continuous process (Wu et al., 2014). The SARS-CoV-2 nucleocapsid protein is a multifunctional protein with potential primary functions of binding to the viral RNA genome and packing it into a long helical nucleocapsid structure or RNP complex (Masters and Sturman, 1990; McBride et al., 2014). Recent systematic proteomic results indicate that SARS-CoV-2 nucleocapsid proteins expressed in HEK293T/17 cells associate with host mRNA binding proteins and stress granule proteins using affinity purification mass spectrometry (AP-MS) with little bound RNA information (Gordon et al., 2020). Studies have investigated several cis-regulating elements and trans-regulating factors involved in discontinuous transcription processes(Sola et al., 2011), but the molecular mechanisms of the nucleocapsid protein involved in this process in SARS-CoV-2 remain unclear.

In previous work, we solved the crystal structure of the nucleocapsid N-terminal RNA binding domain, suggesting a potential leading compound binding pocket in antiviral agent screening applications (Kang et al., 2020). Herein, we continued our work on the structural studies of the nucleocapsid protein by solving the crystal structure of the nucleocapsid protein C-terminal domain (termed as N-CTD) at a resolution of 2.0 Å. By combining structural comparisons and *in vitro* interacting assays, we sought to investigate the potential molecular mechanisms of interplay between the N-CTD and the conserved SARS-CoV-2 TRS and to provide detailed insight into the function of the nucleocapsid protein in discontinuous transcription.

## Materials and Methods

### Cloning, Expression, and Purification

The SARS-CoV-2 N-FL plasmid was a gift from Guangdong Medical Laboratory Animal Center. We designed several constructs, including the SARS-CoV-2 N-CTD domain (residues 250 to 365), SARS-CoV-2 CTD+IDR domain (residues 250 to 419), and SARS-CoV-2 LKR+CTD domain (residues 183 to 365), which were designed using secondary structure predictions and sequence conservation characteristics. The above gene fragments were obtained by PCR. These sequences were then cloned into the pRSF-Duet-1 vector with an N-terminal 6xHis-SUMO tag using BamH1 and Xho1. All the constructs were expressed in the *E. coli Rosetta* strain. When the OD_600_ of the culture reached 0.8–1.0, IPTG (final concentration of 0.1 mM) was used to induce the expression for 18–20 h at 16°C. The culture was collected by a Beckman high-speed centrifuge at 4,500 rpm for 15 min and disrupted by ultrahigh pressure treatment, and the supernatant was separated by centrifugation at 18,000 rpm for 90 min. After nickel column chromatography, followed by Ulp1 protease digestion for tag removal, the SARS-CoV-2 N-CTD proteins were further purified with size-exclusion chromatography (with a buffer consisting of 20 mM Tris-HCl (pH 8.0), 150 mM sodium chloride, and 1 mM dithiothreitol) and then concentrated by ultrafiltration to a final concentration of 45, 0.965, and 4.7 mg/mL. The SARS-CoV-2 N-CTD-S327C and SARS-CoV-2 N-CTD-S289C mutants were constructed using designed primers and PCR and purified with the same protocol as the wild-type N-CTD protein. Details of all oligonucleotide sequences are available from the authors upon request.

### Crystallization and Data Collection

Crystals were grown from a solution containing 100 mM CHES (pH = 9.3) (Hampton research: HR2-256) and 40% PEG6000 (Sigma-Aldrich) by the hanging drop vapor diffusion method at 16°C. Crystals were frozen in liquid nitrogen in reservoir solutions supplemented without a cryoprotectant. X-ray diffraction data were collected at the South China Sea Institute of Oceanology, Chinese Academy of Sciences with the Rigaku X-ray diffraction (XRD) instrument XtaLAB P200 007HF. The structure of SARS-CoV-2 N-CTD was determined by molecular replacement using the SARS-CoV N-CTD structure (PDB ID: 2GIB) as the search model (Saikatendu et al., 2007) with the PHENIX software suite. The X-ray diffraction and structure refinement statistics are summarized in Table 1.

**Table 1:** Data collection and refinement statistics.

**Item**	SARS-CoV-2 N-CTD^*^
Protein Data Bank ID	7DE1
Wavelength (Å)	1.5418
Resolution range	18.48–2.0 (2.071–2.0)
Space group	*P* 2_1_ 2_1_ 2
Unit cell	
a, b, c (Å)	45.214, 101.438, 59.5261
α, β, γ (°)	90, 90, 90
Total reflections	67,575 (6134)
Unique reflections	19,027 (1805)
Multiplicity	3.6 (3.4)
Completeness (%)	99.27 (96.27)
Mean I/sigma (I)	18.58 (6.46)
Wilson B-factor	15.13
R-merge^**^	0.0588 (0.2126)
R-meas^***^	0.06906 (0.2513)
R-pim^****^	0.03568 (0.132)
CC1/2	0.997 (0.921)
CC	0.999 (0.979)
Reflections used in refinement	19,026 (1806)
Reflections used for R-free	1,903 (180)
R-work^#^	0.1744 (0.1961)
R-free^##^	0.2221 (0.2925)
CC (work)	0.954 (0.893)
CC (free)	0.918 (0.842)
Number of non-hydrogen atoms	2,031
Macromolecules	1,775
Solvent	242
Protein residues	223
RMS (bonds) (Å)	0.010
RMS (angles) (°)	1.01
Ramachandran favored (%)	98.17
Ramachandran allowed (%)	1.83
Ramachandran outliers (%)	0.00
Rotamer outliers (%)	0.54
Clashscore	8.75
Average B-factor	16.39
Macromolecules	15.33
Solvent	23.57

* *Statistics for the highest-resolution shell are shown in parentheses*.

** *Rmerge = ∑_hkl_ ∑_i_ |I_i_ (hkl)- < I(hkl) >|/∑_hkl_∑_i_ I_i_(hkl), where I_i_(hkl) is the intensity measured for the ith reflection and < I(hkl) > is the average intensity of all reflections with indices hkl*.

*** *Rmeas, redundancy-independent (multiplicity-weighted) Rmerge (Evans, 2006, 2011)*.

**** *Rpim, precision-indicating (multiplicity-weighted) Rmerge(Diederichs and Karplus, 1997; Weiss, 2001)*.

# *R-work = ∑_hkl_ ||F_obs_ (hkl) |-|F_calc_ (hkl) ||/∑_hkl_ |F_obs_(hkl) |*.

## *R-free is calculated in an identical manner using 10% of randomly selected reflections that were not included in the refinement*.

### Biolayer Interferometry Assays

Biolayer interferometry (BLI) experiments were performed using an Octet RED96e instrument from ForteBio. All assays were run at 25°C with continuous shaking at 100 rpm. PBS with 0.02% Tween 20 was used as the assay buffer. For RNA binding assays, we designed and synthesized six RNA oligomers with following sequences: RNA1, 5' biotin-ACGAAC-3'; RNA2, 5' biotin-AAACGAAC-3'; RNA3, 5' biotin-AAACGAACUU-3'; RNA4, 5' biotin-GUUCGU-3'; RNA5, 5' biotin-GUUCGUUU-3'; and RNA6, 5' biotin-AAGUUCGUUU-3'. All RNA oligomers are dissolved in enzyme-free water. The paired or impaired duplexes of TRSs were obtained with a ratio of 1:1 via the process of heating and annealing. Duplex TRS-1 was obtained by an annealing process of RNA1 with RNA4; Duplex TRS-2 was obtained by an annealing process of RNA2 with RNA5. Duplex TRS-3 was obtained by an annealing process of RNA3 with RNA6. Unpaired Duplex TRS1 was obtained by an annealing process of RNA1 with RNA5; Unpaired Duplex TRS2 was obtained by an annealing process of RNA2 with RNA4. Biotinylated RNA was tethered on super streptavidin (SSA) biosensors (ForteBio) by dipping sensors into 100 nmol RNA solution. Average saturation response levels of 0.8 nm were achieved in 1 min for all samples. Sensors with tethered RNA were washed in assay buffer for 10 min to eliminate non-specifically bound protein molecules and to establish stable baselines before starting association-dissociation cycles with different concentrations of CTD proteins. The collected raw kinetic data were processed in the data analysis software provided by the manufacturer using double reference subtraction in which both the 0.02% Tween-20-only reference and the inactive reference were subtracted. The resulting data were analyzed based on a 1:1 binding model from which K_on_ and K_off_ values were obtained, and then the *Kd* values were calculated.

## Results

### The Crystal Structure of the SARS-CoV-2 Nucleocapsid C-Terminal Domain Reveals a Conserved Stable Dimer Formation Mechanism

**Figure 1:**
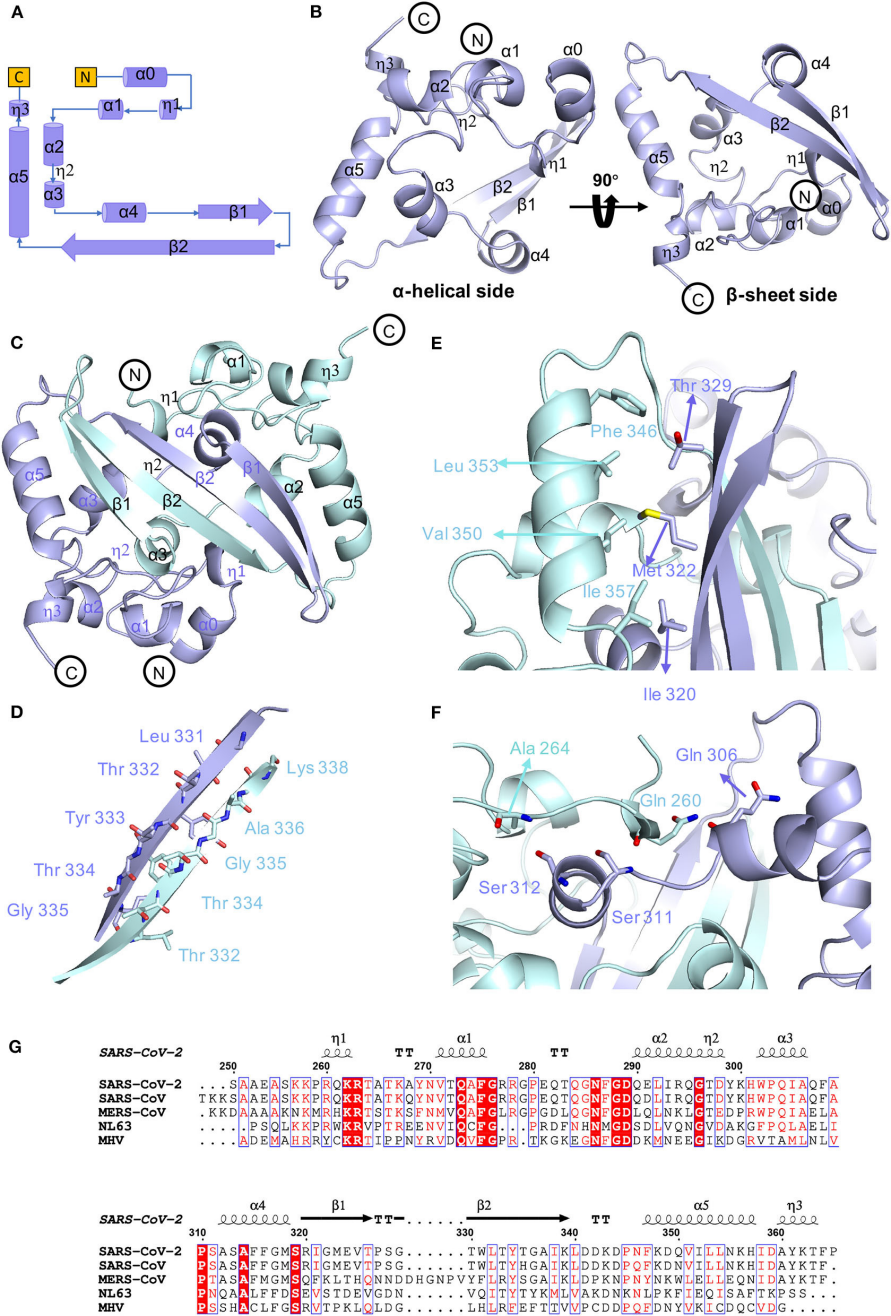
Conserved stable dimer of the C-terminal domain of the SARS-CoV-2 nucleocapsid protein. **(A)** Topological style illustration of the SARS-CoV-2 N-CTD structure. **(B)** Monomer structure of SARS-CoV-2 N-CTD. **(C)** The interaction between SARS-CoV-2 N-CTD monomers. **(D)** The hydrogen bond interactions between SARS-CoV-2 N-CTD monomer B chains. **(E,F)** The hydrophobic interactions between SARS-CoV-2 N-CTD molecules. **(G)** Sequence alignment of SARS-CoV-2 N-CTD, SARS-CoV N-CTD (UniProtKB: P59595), MERS-CoV N-CTD (UniProtKB: R9UM87), HCoV-NL63 N-CTD (UniProtKB: P33469), and MHV N-CTD (UniProtKB: NP_040838.1). Red arrows indicate conserved residues for ribonucleotide binding sites, and dashed boxes indicate variable residues in the structural comparisons.

To determine the precise structural information of the novel coronavirus SARS-CoV-2 N-CTD, we solved the N-CTD structure at a resolution of 2.0 Å with X-ray crystallography. The final structure was refined to R-factor and R-free values of 0.1744 and 0.2221, respectively. The complete statistics for the data collection, phasing, and refinement are presented in Table 1. One N-CTD monomer is composed of three 3_10_-helices, five α-helices, and two β-strands (Figure 1A), with an additional N-terminal α_0_-helix in the partial electron density of the traced molecule. And SARS-CoV-2 N-CTD consists of two “C-shaped” monomers to form a stable dimer (Figures 1A,B). As shown in Figure 1C, two N-CTD monomers utilize three regions to form a stable symmetrical dimer, with a buried surface area of 2618 Å^2^ (of the 8026 Å^2^ monomer surface) (calculated by the online PISA server). First, the most distinctive feature is the antiparallel four-stranded β-sheet that has domain swapping interactions between the two monomers. Within the β-sheet, β2 strands of monomers interact with each other via a wide range of stable hydrogen bonds (Figure 1D). Second, residues F346, L353, V350, and I357 of the longest α5-helix form multiple intermolecular hydrophobic interactions with residues T329, M322, and I320 in the β1-strand (Figure 1E). Last, the α1 helix forms a strong hydrophobic interaction with other monomer α4 helixes, like two clip-bars, to firmly fix the two monomers (i.e., A264-S312, Q260-S311, and Q260-Q306 interactions, as shown in Figure 1F). Further gel filtration results also support the dimerization of N-CTD in solution (Supplementary Figure 1B). Therefore, the stable dimerization of SARS-CoV-2 N-CTD shows a conserved stable dimerization status in both crystal packing and solution conditions.

As shown in the sequence alignments of betacoronaviruses, the amino acid sequences of the SARS-CoV-2 N-CTD and the counterpart proteins of the highly pathogenic SARS-CoV and MERS-CoV and the low pathogenic HCoV-NL63 were quite different, with sequence identities of 89.74, 48.59, and 35.71% (Figure 1G), respectively. However, the overall structure of SARS-CoV-2 N-CTD is similar to the N-CTD of previously reported coronaviruses (including SARS-CoV; Yu et al., 2006; Chen et al., 2007; Takeda et al., 2008, HCoV-NL63; Szelazek et al., 2017, MERS-CoV; Nguyen et al., 2019, mouse hepatitis virus (MHV); Ma et al., 2010, and infectious bronchitis virus (IBV); Jayaram et al., 2006).

### Potential Self-Interactions of the SARS-CoV-2 N-CTD Dimer

**Figure 2:**
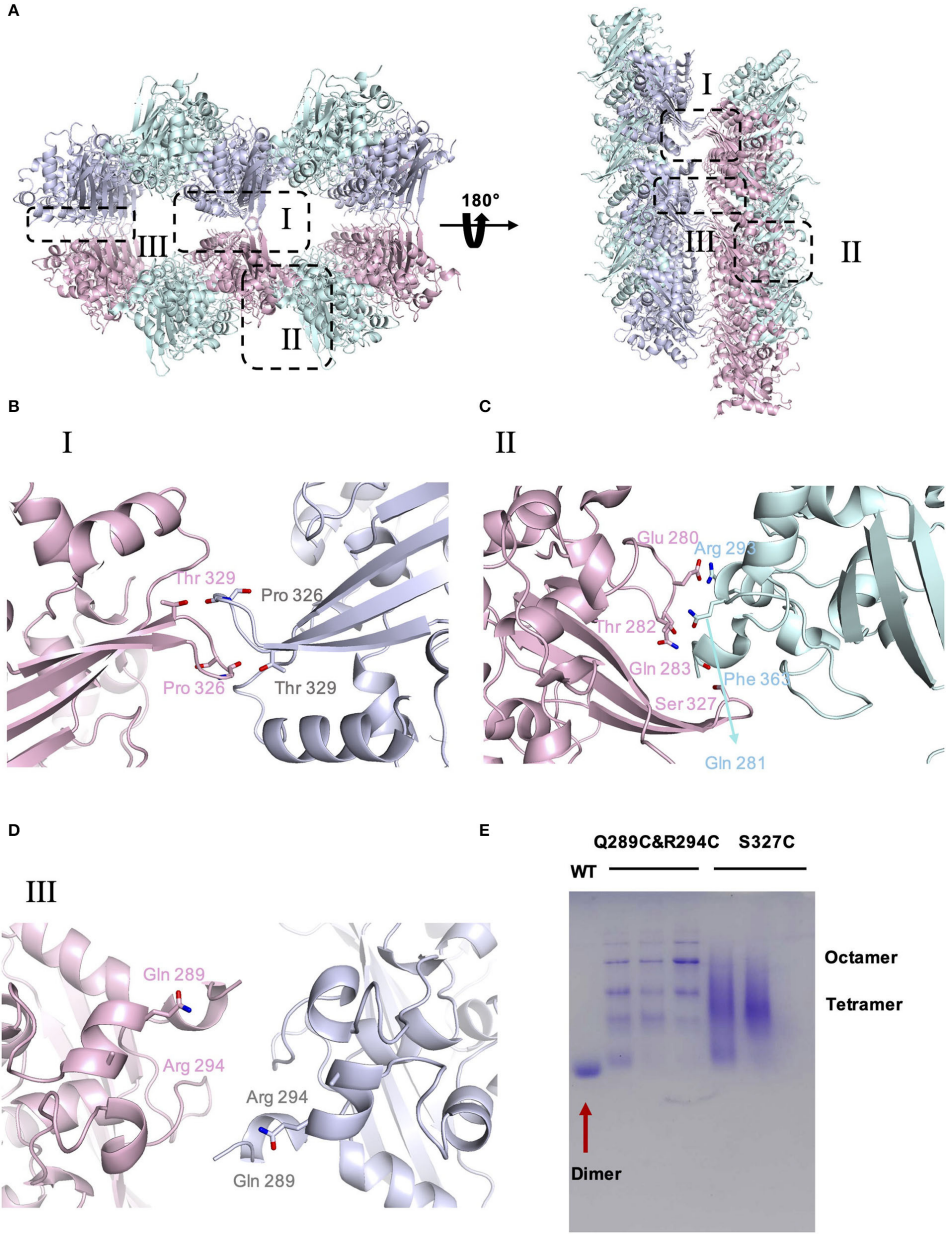
The crystal packing of SARS-CoV-2 N-CTD. **(A)** SARS-CoV-2 N-CTD high-order oligomers in the crystal packing. The interaction regions of putative high-order oligomers are highlighted with dashed boxes I, II, and III. **(B)** Zoomed-in detailed view of the interaction of symmetric molecules in dashed box I. **(C)** Zoomed-in detailed view of the interaction of symmetric molecules in dashed box II. **(D)** Zoomed-in detailed view of the interaction of symmetric molecules in dashed box III. **(E)** Native PAGE analysis of SARS-CoV-2-N-CTD and mutants in disulfide trapping assays.

To investigate the potential self-interactions of SARS-CoV-2 N-CTD, we next analyzed the symmetry molecules of crystal packing. As shown in Figure 2A, SARS-CoV-2 N-CTDs form repeating cylindrical high-order structures with six dimers, which is slightly different from the SARS-CoV N-CTD octamer X-shaped high-order oligomer pattern. There are three key features of the SARS-CoV-2 N-CTD's potential self-interactions in our study (Figure 2A). To validate these potential self-interaction features in solution, we performed *in vitro* disulfide trapping assays by engineering single-site cysteine mutations at the feature I, II, and III regions. Since wild-type SARS-CoV-2 N-CTD does not contain any cysteine residues, the exotic cysteine residues will form disulfide bonds within suitable distances (Bass et al., 2007). The first remarkable feature is that residues P326 and T329 in the β5–β6 loop interact with symmetric molecules in the same position, forming a hand-in-hand-like structure (Figure 2B). The second loop is another β5–β6 loop of the same N-CTD dimer that interacts with the α2-helix and C-terminal tail of the other side of the symmetric molecule via salt bridges or hydrogen bonds (Figure 2C). As shown in Figures 2B,C, S327 is located at a favorable position of the β5–β6 loop, which mediates self-interactions within the feature I and II regions. The last feature is a salt bridge interaction between the side chains of Q289 and R294 (Figure 2D). Q289 and R294 are located at the α1-helix region, which mediates self-interactions within the feature III region. Therefore, the S327C single mutation and the Q289C with R294C double mutation were expressed and tested through size-exclusion chromatography. As shown in Figure 2E, compared with the wild-type SARS-CoV-2 N-CTD, the positions of the S237C and Q289C/R294C mutant proteins shifted up to varying degrees, and these mutants are in a higher polymerization state than the dimer. These results suggest that SARS-CoV-2 N-CTD has potential self-interactions via the β5–β6 loop and α1-helix regions. In summary, the β5–β6 loop and α1-helix regions are important for the self-association of SARS-CoV-2 N-CTD in the crystal packing.

### Surface Electrostatic Potential Characteristics of SARS-CoV-2 N-CTD

**Figure 3:**
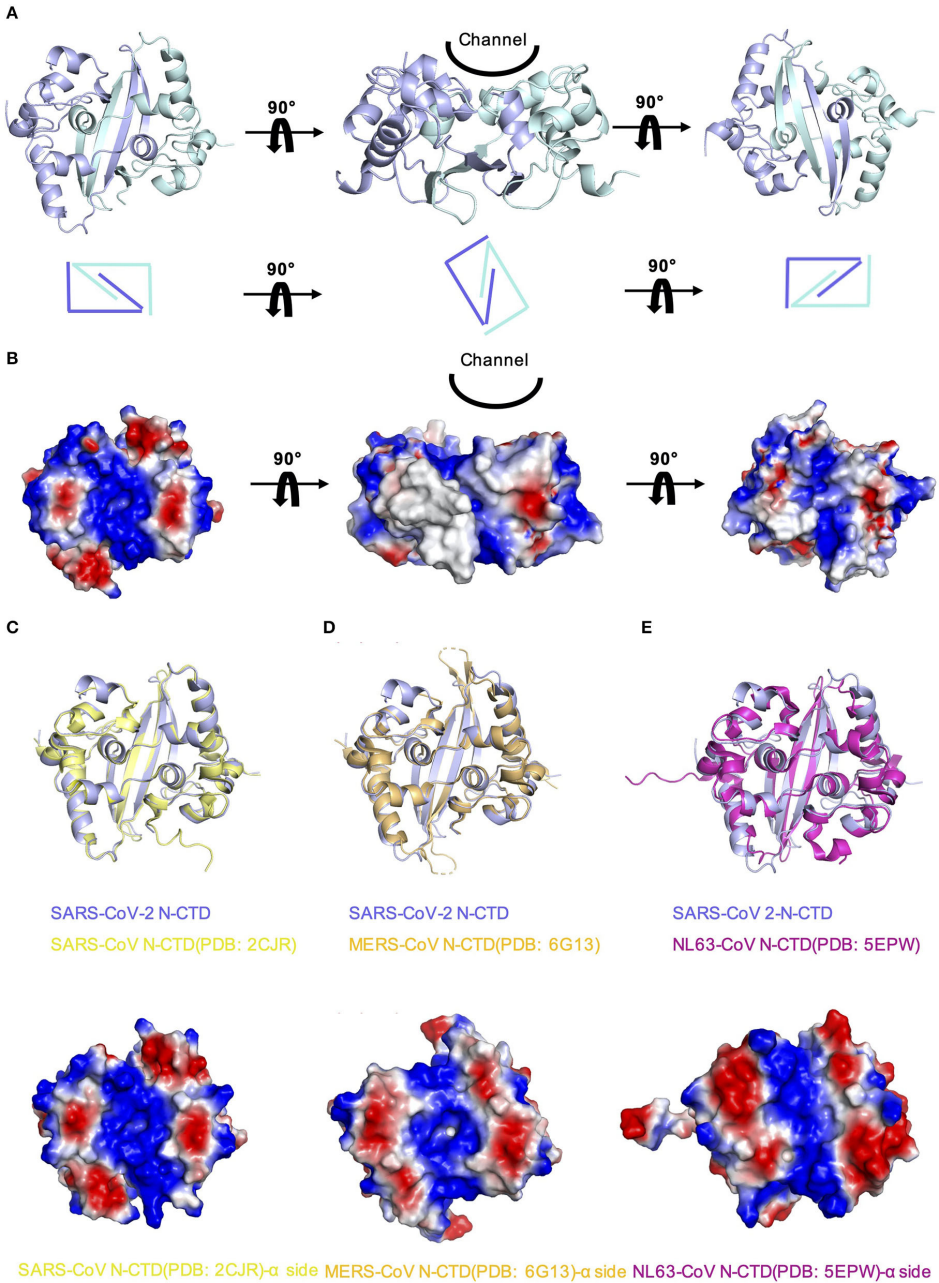
Comparison of SARS-CoV-2 N-CTD with related viral N-CTD structures. **(A)** Structure of SARS-CoV-2 N-CTD. **(B)** Electrostatic surface of SARS-CoV-2 N-CTD. Blue denotes a positive charge potential, while red indicates a negative charge potential. **(C)** Overall structural comparison of SARS-CoV-2 N-CTD with SARS-CoV N-CTD. Top panel: superimposition of SARS-CoV-2 N-CTD (blue) with SARS-CoV N-CTD (yellow). Bottom panel: electrostatic surface of SARS-CoV N-CTD. **(D)** Overall structural comparison of SARS-CoV-2 N-CTD with MERS-CoV N-CTD. Top panel: superimposition of SARS-CoV-2 N-CTD (blue) with MERS-CoV N-CTD (orange). Bottom panel: electrostatic surface of MERS-CoV N-CTD. **(E)** Overall structural comparison of SARS-CoV-2 N-CTD with CoV-NL63 N-CTD. Top panel: superimposition of SARS-CoV-2 N-CTD (blue) with SARS-CoV N-CTD (magenta). Bottom panel: electrostatic surface of the CoV-NL63 N-CTD.

In order to explore whether there are other RNA binding domains that exist in the rest of the nucleocapsid in SARS-CoV-2, we analyzed the surface electrostatic potential characteristics of the SARS-CoV-2 N-CTD based on the structure. The dimer of SARS-CoV-2 N-CTD is shown as a cuboid shape, as described above, containing an α-helix-rich side and a β-sheet side (Figure 3A). The electrostatic surface suggests that the α-helix-rich side contains a positively charged channel (Figure 3B, the left panel), whereas the β-sheet side is a neutral surface (Figure 3B, the right panel). Its shape is the same as the positively charged channel rich in α-helix side in SARS-CoV and MERS-CoV (Figures 3C,D). Intriguingly, although low pathogenic CoV-NL63 has a similarly positively charged channel in its nucleocapsid CTD, the shape of the channel is quite different (Figure 3E) (Szelazek et al., 2017). The surface charge of another low pathogenic IBV is also different from that of SARS-CoV-2 (Supplementary Figure 2) (Jayaram et al., 2006). Nevertheless, the conserved positively charged channel of the α-helix-rich side is thought to be a potential RNA binding site in the SARS-CoV-2 N-CTD.

### SARS-CoV-2 N-CTD With Flanking Regions Recognizes Transcriptional Regulatory Sequences

**Figure 4:**
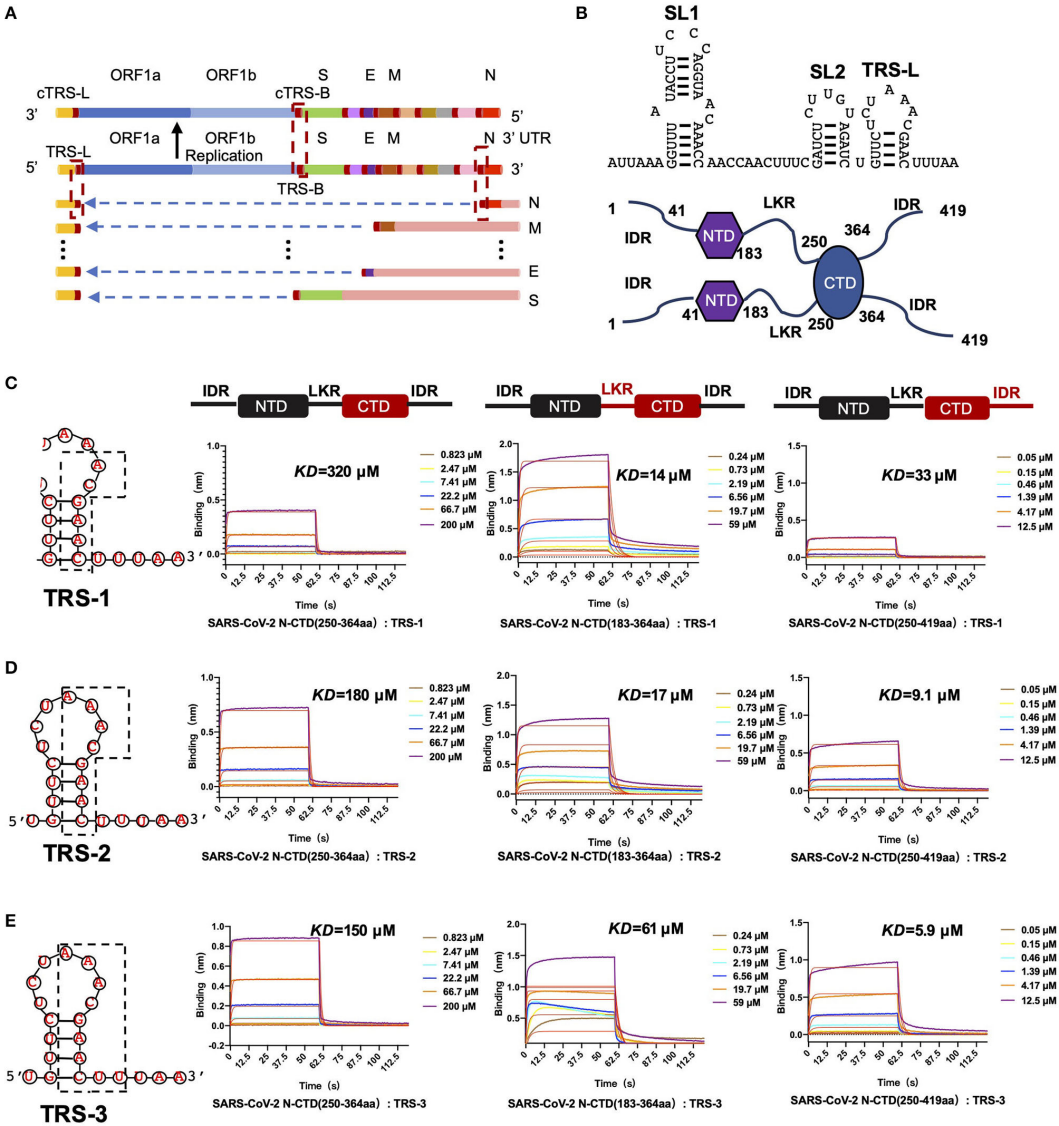
SARS-CoV-2 N-CTD with flanking regions recognizes transcriptional regulatory sequences (TRSs). **(A)** Schematic diagram of TRS distribution in the SARS-CoV-2 genome. TRS-L, TRS in the preamble; ORF, open reading frame; S, spike glycoprotein-encoding region; E, envelope protein-encoding region; M, membrane protein-encoding region; N, nucleocapsid protein-encoding region. **(B)** Top panel: the secondary structure of 5' leader region sequences. SL1: The first neck ring structure. SL2: The second neck ring structure. Bottom panel: the dimerization form of N-CTD in solution. **(C)** The interaction of TRS-1 with SARS-CoV-2 N-CTD, N-(LKR+CTD), and N-(CTD+IDR). Left: TRS-1 in TRS-L. **(D)** The interaction of TRS-2 with SARS-CoV-2 N-CTD, N-(LKR+CTD), and N-(CTD+IDR). Left: TRS-2 in TRS-L. **(E)** The interaction of TRS-3 with SARS-CoV-2 N-CTD, N-(LKR+CTD), and N-(CTD+IDR). Left: TRS-3 in TRS-L.

We hypothesize that SARS-CoV-2 N-CTD is capable of binding to viral RNA, especially the most conserved transcriptional regulatory sequences (TRSs) of the viral genome. Recent genomic data suggested that there are 10 TRSs in the SARS-CoV-2 genome, with one TRS in the 5′ leader region (TRS-L) and nine TRSs in the 3′ region (TRS-B) of the genome (Kim et al., 2020) (Figure 4A). To study the mechanisms of SARS-CoV-2 N-CTD protein recognition of TRS, we next synthesized three TRSs in the leader region for *in vitro* binding assays, named TRS-1 (5′-ACGAAC-3′, 6 nucleotides), TRS-2 (5′-AAACGAAC-3′, 8 nucleotides), and TRS-3 (5′-AAACGAACUU-3′, 10 nucleotides) (Figure 4B). For the shortest sequence, TRS-1, SARS-CoV-2 N-CTD showed a very weak binding affinity of 320 μM via biolayer interferometry assays (Figure 4C, the left panel). However, SARS-CoV-2 N-CTD with a flanking internal disorder region, regardless of the middle LKR motif (residues 183 to 365, termed LKR+CTD) or the C-terminal IDR (residues 250 to 419, termed CTD+IDR), showed up to a 20-fold elevated binding affinity with *Kd* values of 14 and 33 μM, respectively (Figure 4C, the middle and right panels). As the length of the TRS increased, the binding affinities were enhanced (Figures 4D,E). TRS-2 interacted with CTD, CTD+IDR, and LKR+CTD with binding affinities of 180, 9.1, and 17 μM, respectively. Furthermore, we found that the tightest interaction is CTD+IDR bound to TRS-3, with a *Kd* value of 5.9 μM (Figure 4E, right panel), whereas CTD and LKR+CTD bind to TRS-3 with a *Kd* value of 150 and 61 μM, respectively.

**Figure 5:**
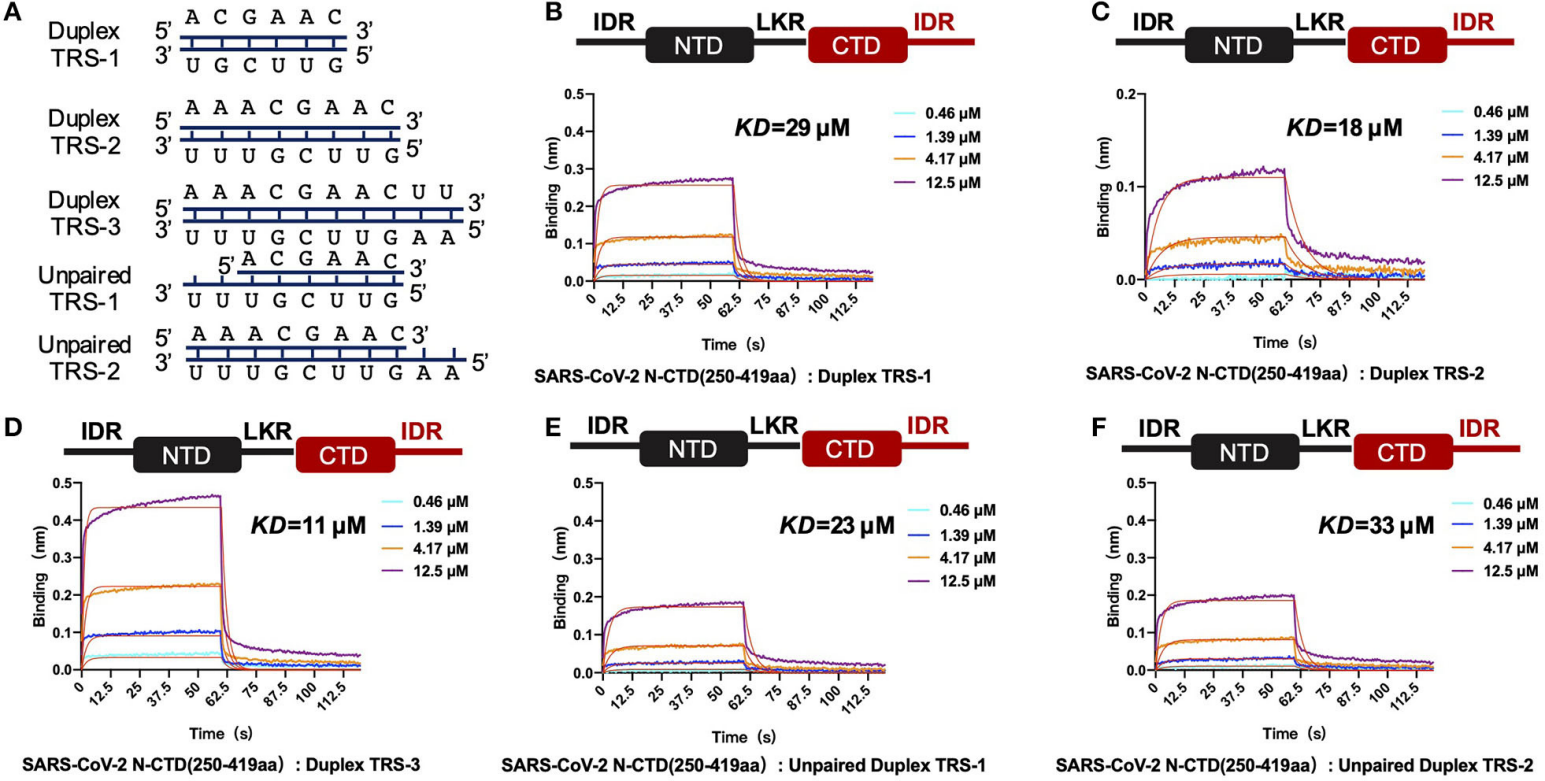
SARS-CoV-2 N-CTD with C-terminal IDR regions recognizes paired and unpaired transcriptional regulatory sequences (TRSs). **(A)** Schematic diagram of the paired double-stranded RNAs (Duplex TRSs) and imperfectly unpaired double-stranded RNAs (unpaired TRSs). **(B)** The interaction of duplex TRS-1 with SARS-CoV-2 N-(CTD+IDR). **(C)** The interaction of duplex TRS-2 with SARS-CoV-2 N-(CTD+IDR). **(D)** The interaction of duplex TRS-3 with SARS-CoV-2 N-(CTD+IDR). **(E)** The interaction of imperfectly paired duplex TRS-1 with SARS-CoV-2 N-(CTD+IDR). **(F)** The interaction of imperfectly paired duplex TRS-2 with SARS-CoV-2 N-(CTD+IDR).

In the single positive strand of the viral RNA genome, TRS-L has a stem-loop structure. Along with the discontinuous transcription process, the TRS has two other states: the single-stranded TRS-B and the double-stranded fully paired cTRS (Figure 5A). Therefore, we next continued to explore if there are any differences in CTD+IDR with different TRSs. The CTD+IDR protein binds to Duplex TRS-1 (TRS1-cTRS1, 6 bp), Duplex TRS-2 (TRS2-cTRS2, 8 bp), and Duplex TRS-3 (TRS3-cTRS3,10 bp) with double-stranded TRS-paired RNA with *Kd* values of 29, 18, and 11 μM, respectively (Figures 5B–D). The binding affinities are approximately or slightly weaker than those of its single-stranded counterpart. For the imperfectly paired RNA composed of TRS1-cTRS2(unpaired TRS-1) or TRS2-cTRS-3(unpaired TRS-2), the binding affinities were similar to those of double-stranded RNA substrates, with *Kd* values of 23 and 33 μM, respectively (Figures 5E,F). To summarize the binding results, we determined that the unpaired adeno dinucleotides in the 5′ regions of the TRS, which exist in TRS-3 and cTRS-3 but not in the paired RNA or imperfectly paired RNA substrates, have a micro-molar binding affinity to nucleocapsid protein CTD-IDR constructs, whereas the unpaired adeno dinucleotides in the 3′ region of the TRS (i.e., inside the TRS2-cTRS-3 imperfectly paired RNA) have a weaker binding ability to the same protein. Combined with the TRS-L model, these data suggest that the 5′ unpaired adeno dinucleotide in the stem-loop region of TRS-L is a key factor involved in the binding of the nucleocapsid protein.

## Discussion

The N protein mediates ribonucleoprotein (RNP) complex formation via two key steps: packaging of the viral RNA genome and self-assembly of oligomerizations. Studies on coronavirus N-CTD suggest that the multiple packing modes of N-CTD dimers probably lead to the formation of rigid helically symmetric nucleocapsids, an unusual feature that is supported by various biochemical assays, including the disulfide trapping technique (Jayaram et al., 2006; Chen et al., 2007; Chang et al., 2013; Gui et al., 2017). Currently, the SARS-CoV N-CTD domain self-association has been widely studied for viral RNP assembly (Surjit et al., 2004; Yu et al., 2005; Luo et al., 2006). However, the role of N-CTD in the self-association of SARS-CoV-2 remains unclear. Our structural data suggest that SARS-CoV-2 N-CTD possesses conserved dimerization mechanisms via multiple hydrophilic and hydrophobic interactions, similar to the CTD of other coronavirus nucleocapsid proteins. Intriguingly, the higher-order self-association of SARS-CoV-2 N-CTD seems different from that of SARS-CoV N-CTD in our studies. Previous studies showed that SARS-CoV N-CTD packs into octamers and forms a twin helix in the crystal packing (Chang et al., 2014); however, SARS-CoV-2 N-CTD packs into a cylindrical shape in the crystal packing. To further verify these observations, *in vitro* disulfide trapping assays combined with size-exclusion chromatography were performed to illustrate the status of SARS-CoV-2 N-CTD in solution. Our data suggest that the observed potential self-interactions via the β5–β6 loop and α1-helix regions in the crystal actually exist in solution, which may serve as the first step of the RNP assembly process.

Previous studies suggest that the coronavirus nucleocapsid contains multiple RNA binding sites, including the NTD, CTD, and C-terminal IDR regions (Chang et al., 2014). Our previous work demonstrated that the N-terminal domain of the nucleocapsid is capable of binding to viral single-stranded 32-mer RNA. Our structural data suggest that SARS-CoV-2 N-CTD contains a positively charged channel similar to MERS-CoV N-CTD and SARS-CoV N-CTD. These surface electrostatic potential characteristics are conserved among the highly pathogenic viral nucleocapsid proteins (Chen et al., 2007; Nguyen et al., 2019). These positively charged channels in the α-helix-rich side are considered as potential RNA binding sites in SARS-CoV-2 N-CTD.

Previous studies demonstrated the role of the CTD in the recognition of the packaging signal in coronavirus nucleocapsid proteins in CoVs, such as MHV (Kuo et al., 2014), MERS-CoV (Hsin et al., 2018), and SARS-CoV (Chang et al., 2009), but HCoV-NL63 N-CTD fails to bind RNA (Zuwała et al., 2015). The nucleocapsid proteins of coronaviruses are homologous and possess a conserved modular composition comprising five domains, represented as the N-tail domain, NTD, LKR, CTD, and C-IDR. The N-NTD, N-CTD, and C-IDR were all reported to bind viral RNA in SARS-CoV (Huang et al., 2004; Chen et al., 2007; Takeda et al., 2008). However, the roles of these domains in RNA binding remain to be elucidated in the SARS-CoV-2 N protein. Our early work suggests that SARS-CoV-2 N-NTD displays a modest binding affinity to viral transcriptional regulatory sequence (TRS) RNA, with a *Kd* value of 140 μM (Kang et al., 2020). In our study, although SARS-CoV-2 N-CTD binds to TRS RNA with a relatively weaker binding affinity (*Kd* value of 320 μM), SARS-CoV-2 N-CTD with flanking regions (either LKR or C-IDR) demonstrated interactions with the same RNA template in a micro-molar binding affinity(the highest *Kd* value was 5.9 μM). The flanking regions of CTD are rich in positively charged amino acids (seven arginines and four lysines out of 69 total residues in the N-terminal flanking region, with one arginine and nine lysines out of 55 residues in C-terminal flanking region.). These characteristics of the SARS-CoV-2 N-CTD may explain how the franking regions are beneficial to the binding of RNA. To our knowledge, the function of the SARS-CoV-2 N LKR motif, which enhances CTD binding to the TRS RNA sequence beyond its potential phosphorylation function of oligomerization (Peng et al., 2008), is reported here for the first time.

In conclusion, in this paper, we analyzed the crystal structure of the nucleocapsid C-terminal domain, studied the potential self-interaction formation of SARS-CoV-2 N-CTD, and verified the self-interaction characteristics of the single-point mutant. By studying the recognition mechanism of SARS-CoV-2 N-CTD protein to TRS, it is found that the 5′ unpaired adeno dinucleotide in the stem-loop region of TRS-L is a key factor involved in the binding of nucleocapsid protein. Altogether, these results reveal a new method of viral transcription sequences mechanism.

## Data Availability Statement

The datasets presented in this study can be found in online repositories. The names of the repository/repositories and accession number(s) can be found at: Protein Data Bank, 7DE1.

## Author Contributions

SC and SK contributed to the conception of the study and performed the structural determination and validation. SC, SK, and MY constructed the article, designed the experiments, created the figures, and wrote the manuscript. SK, MY, and SH performed the protein purification, crystallization, *in vitro* protein-protein interaction, and biochemical experiments. XC, ZH, ZiZ, ZhZ, and QC assisted in analyzing the experimental results of the protein-protein interactions. All authors contributed to the article and approved the submitted version.

## Conflict of Interest

The authors declare that the research was conducted in the absence of any commercial or financial relationships that could be construed as a potential conflict of interest.
