# Supplementary material for: Structural Insight Into the SARS-CoV-2 Nucleocapsid Protein C-Terminal Domain Reveals a Novel Recognition Mechanism for Viral Transcriptional Regulatory Sequences
Source: Front Chem. 2021 Jan 12;8:624765. doi: 10.3389/fchem.2020.624765 (PMC7835709; doi:10.3389/fchem.2020.624765)
Supplement: Supplementary file 1 [file Table_1.DOCX]

Supplementary Material

# Supplementary Figures

**Supplementary Figure 1.** (**A**) The structural model of SARS-CoV-2 nucleocapsid protein. (**B**) The dimerization form of N-CTD in solution.

**Supplementary Figure 2.** Electrostatic surface of the IBV N-CTD. Blue denotes positive charge potential, while red indicates negative charge potential.
